# Community-oriented collaboration for mental health care and mental health promotion

**DOI:** 10.1080/17571472.2015.1136127

**Published:** 2016-02-06

**Authors:** Paul Thomas, David Morris

**Affiliations:** ^a^ GP, West London, Editor London Journal of Primary Care; ^b^ Professor of Mental Health, Inclusion and Community, University of Central Lancashire, Editor London Journal of Primary Care

In 2015 LJPC convened an expert panel, in partnership with Royal College of General Practitioners (RCGP) and the Educational Trust for Health Improvement through Cognitive Strategies (the ETHICS Foundation). The panel produced three documents to assist thinking about practical ways to integrate primary mental health care and mental health promotion. These can be downloaded free from: http://ethicsfoundation.org/2015/09/04/mental-health-promotion-saves-lives/.

In this LJPC Issue, the first of 2016, Steve Thomas presents the 12 recommendations arising from the Expert Panel. These recommendations are being moved forward by the RCGP.

Also in this issue is an important and sensitive paper by Robert Winston and Rebecca Chicot that explains how love in infancy leads to good mental health later on. The science of epigenetics reveals rapid brain growth and circuitry in early years – estimated at an astonishing 700–1000 synapse connections per second. Those circuits shape what happens later on. Without a good initial bond, children are less likely to grow up to become happy, independent adults. The paper confirms a truth: that hugs, lullabies and smiles from parents really do help babies to become resilient and compassionate adults, even offering inoculation against adolescent angst decades later – and help too in passing their exams.

Amelia Martin continues the theme of resilience and compassion. She describes the Royal Society of Medicine medical humanities conference on the 5th of October 2015 that set out to explore the relationships between literature, the humanities and health care. A key message is that practitioners must *connect* as well as communicate with patients.

Reflecting on a Tate Modern exhibition, Francesco Carelli reflects on how the absence of compassion also harms adults. Malevich was a Russian artist who died prematurely because his dream of a new social order did not fit the vision portrayed by powerful others.

LJPC will continue the theme of collaboration for good mental health throughout 2016. We want to reveal ways to integrate care for mental illness, and also ways to collaborate for good mental health at all stages of life. This requires learning organisations and learning systems and healthy communities, as well as healthy, active, compassionate individuals.

We will publish an account of ‘Connected Communities’, a five-year study in seven sites that reveals the well-being, citizenship, capacity and economic dividends that community capital can generate. We will publish papers on parenting, partnership between schools and clusters of general practices, how music and the arts can contribute to healing and building communities. We will highlight the social and occupational impacts on mental health at all stages of life – parents, schools, workplace, retirement, the third age, end of life. We will argue that primary care does not have to do all of this, but that it is key to orchestrating it. Together we need to define a curriculum of skills and systematic ways for health care practitioners and managers to learn how to do this in sustainable, enjoyable ways.

The central role of primary care in integrating mental health care and mental health promotion was noted over a decade ago by the much-missed Helen Lester, in a British Journal of General Practice paper titled ‘Integrated primary mental health care: threat or opportunity in the new NHS?’.[1] She and her co-authors explained that:

[Primary care] occupies an important space at the interface of users, families, communities, and professional worlds and is able to address mental, physical, and social aspects of care. Primary care is also a low-stigma setting, able to offer rapid access for both routine and crisis care, a longitudinal approach to care where patients are never discharged, and perhaps above all, interpersonal continuity of care …

… Primary care is perhaps better placed to fulfil each of these elements of continuity than secondary care specialist services. Primary care also appears to be generally preferred as a setting by both mental health service users and carers …

This *new localism* is now NHS policy. In the 2014 Five Year Forward View [2] the NHS Chief Executive stated:

The future will see far more care delivered locally but with some services in specialist centres, organised to support people with multiple health conditions, not just single diseases …

It will require teamworking, strategic partnerships and ‘local health communities’ for positive mental health promotion for all citizens, as well as care for those who are mentally unwell:

New partnerships with local communities, local authorities and employers.

Patients will gain far greater control of their own care

NHS will become a better partner with voluntary organisations and local communities.

It will require systematic working across disciplinary and organisational boundaries:

Break down the barriers in how care is provided … between family doctors and hospitals, between physical and mental health, between health and social care.

London Journal of Primary Care wants to engage a broad network of thinkers and activists to advance Lester’s vision for integrated primary mental health care and mental health promotion. The stress inside contemporary general practices is intolerable; the present silo-based way of operating is unsustainable. We have no option but to learn how to orchestrate a broader collaboration for health within which everyone contributes for synergy. You can follow the discussion on the *London Journal of Primary Care* website – http://explore.tandfonline.com/content/med/london-journal-of-primary-care-page where all articles are available for free, and by signing up to our table of contents alerts.

Paul Thomas Paul.thomas7@nhs.net David Morris dmorris1@uclan.ac.uk
